# The neurologic face of X-linked lymphoproliferative syndrome type 1: a systematic review

**DOI:** 10.1186/s13023-025-04057-9

**Published:** 2025-10-21

**Authors:** Erta Rajabi, Saber Gharagozlou, Kousha Farhadi, Moeinadin Safavi, Fatemeh Zamani, Abdolreza Javadi, Zahra Rahemi, Parastoo Rostami, Nima Parvaneh

**Affiliations:** 1 Division of Allergy and Clinical Immunology, Department of Pediatrics, Children’s Medical Center, Tehran University of Medical Sciences, No. 62 Qarib St., Tehran, 1419733152 Iran; 2 Anesthesia, Critical Care, and Pain Management Research Center, Tehran University of Medical Sciences, Tehran, Iran; 3 Department of Pathology, Children’s Medical Center, Tehran University of Medical Sciences, Tehran, Iran; 4 Department of Radiology, Children’s Medical Centre, Tehran University of Medical Sciences, Tehran, Iran; 5 Advanced Diagnostic and Interventional Radiology Research Center, Tehran University of Medical Sciences, Tehran, Iran; 6 Department of Pathology, Imam Hossein Hospital, Shahid Beheshti University of Medical Sciences, Tehran, Iran; 7 Department of Pediatrics, Children’s Medical Center, Tehran University of Medical Sciences, Tehran, Iran

**Keywords:** X-linked lymphoproliferative syndrome, XLP1, *SH2D1A*, Neuroinflammation, Encephalitis, Lymphoma, Vasculitis, Inborn error of immunity, Hematopoietic stem cell transplantation, Systematic review

## Abstract

**Background:**

X-linked lymphoproliferative syndrome type 1 (XLP1) is a rare inborn error of immunity with high mortality rates. Neurological manifestations may be the presenting features and are often fatal; however, their characterization is insufficient, hindering optimal clinical management. The aim of this study is to systematically review the neurological characteristics, outcomes, and survival in XLP1 patients and identify parameters associated with improved prognosis. A PRISMA-guided analysis of PubMed, Web of Science, Scopus, and Embase (up to March 2025) identified studies documenting neurological involvement in genetically verified XLP1 patients. We extracted data on clinical features, neuroimaging findings, therapeutic interventions, and survival.

**Results:**

We identified 42 genetically verified XLP1 patients with neurological involvement. Central nervous system (CNS) involvement comprised hemophagocytic lymphohistiocytosis (HLH) in 38.1%, vasculitis in 28.6%, and lymphoma in 19% of them. The development of brain vasculitis several months after Burkitt’s lymphoma was a specific presentation. The median age of neurological onset was 5 years. The predominant presenting symptoms were seizures (47.6%), altered consciousness (35.7%), and headaches (21.4%). Neuroimaging frequently revealed abnormalities in the temporal lobe and basal ganglia, often with hemorrhage and edema. Epstein-Barr virus (EBV) was identified in 54.8% of cases, sometimes limited to brain tissue. CSF analysis frequently showed elevated protein and pleocytosis. SH2D1A mutations were diverse, with Arg55 and Trp64 identified as recurrent hot spots. The overall mortality reached 52.4%, with most deaths occurring within five years of neurological onset. Conventional immunosuppressive and cytotoxic treatments were largely ineffective in changing the disease course. In exploratory analysis, hematopoietic stem cell transplantation (HSCT) did not significantly improve survival in the primary dataset, although sensitivity analysis suggested a possible benefit.

**Conclusions:**

Neurological involvement in XLP1 exhibits clinical heterogeneity and carries a high mortality rate. Early recognition and timely HSCT may improve survival, demonstrating the importance of vigilant neurological monitoring in affected individuals.

**Supplementary Information:**

The online version contains supplementary material available at 10.1186/s13023-025-04057-9.

## Background

X-linked lymphoproliferative syndrome type 1 (XLP1, OMIM #308240) is an inborn error of immunity (IEI) characterized by an aberrant response to the Epstein-Barr virus (EBV) infection [1]. This disorder arises from mutations in *SH2D1A*, which encodes the signaling lymphocyte activation molecule (SLAM)-associated protein (SAP), an essential cytoplasmic adaptor protein required for the function of T cells and natural killer (NK) cells [2, 3]. SAP deficiency results in impaired cytotoxic T lymphocyte and NK cell responses, leading to inadequate viral clearance and subsequent immune dysregulation [4]. This presents clinically as hemophagocytic lymphohistiocytosis (HLH), hypogammaglobulinemia, and lymphoproliferative disorders, with mortality rates historically reaching 81% [5–7].

While these systemic manifestations are well-defined, neurological involvement in XLP1 remains poorly understood despite its potential severity and impact on prognosis. Involvement of the central nervous system (CNS) may happen independently of EBV infection and can manifest as the initial clinical feature, making early recognition challenging. The neuroinflammatory mechanisms underlying these manifestations likely involve direct viral invasion, immune-mediated vasculitis, and dysregulated inflammatory responses [5, 8–11].

The majority of the knowledge regarding XLP1 neurological manifestations comes from isolated case reports, limiting our understanding of the spectrum, prevalence, and consequences of CNS involvement. This knowledge gap hinders evidence-based clinical decision-making and effective patient management measures.

This systematic review seeks to delineate the range of neurological pathologies and their manifestations in XLP1 as well as to assess clinical outcomes and survival trends.

## Methods

**Fig. 1:**
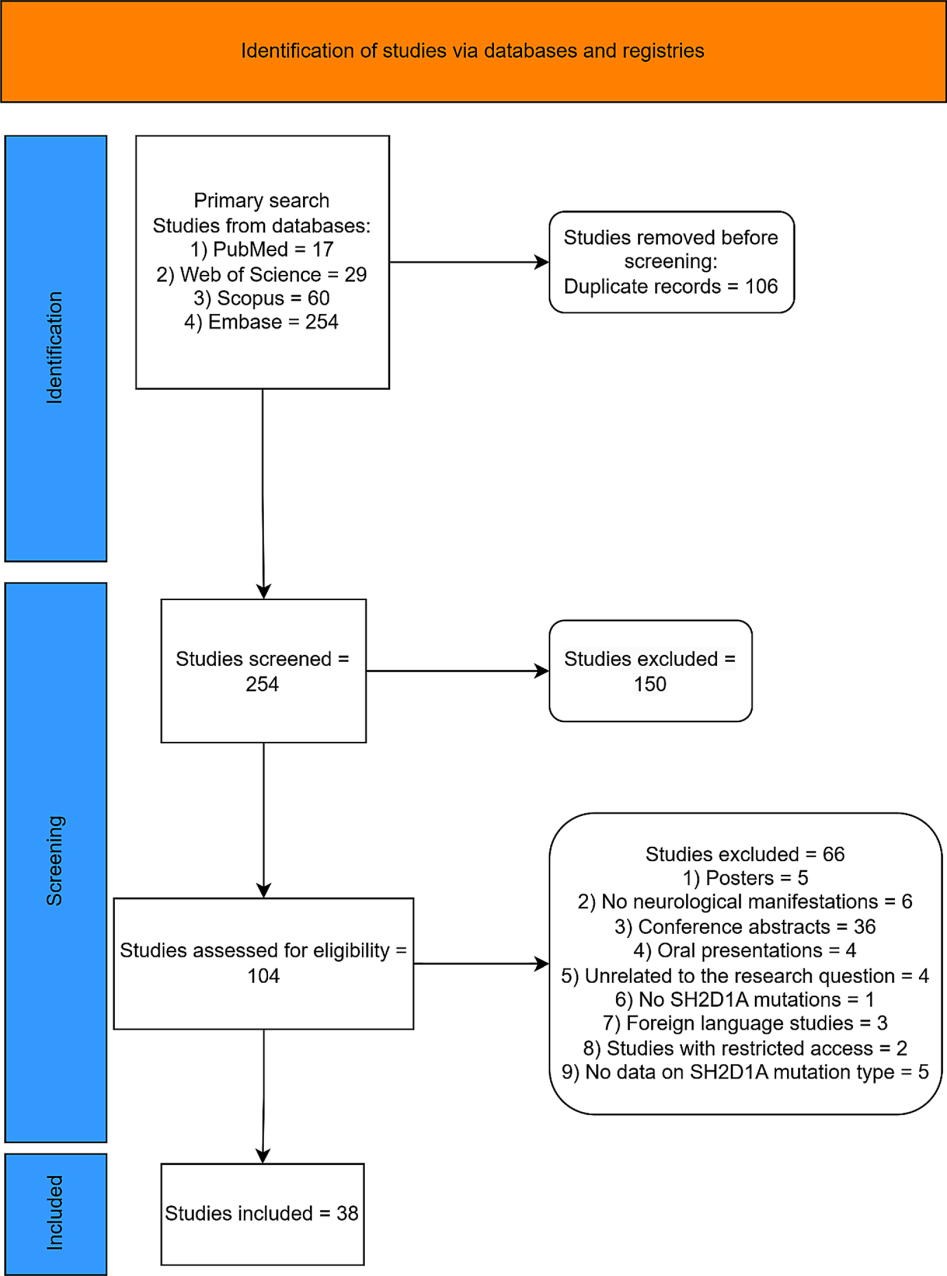
PRISMA flowchart of the study

This systematic review was conducted in accordance with the 2020 guidelines of the Preferred Reporting Items for Systematic Reviews and Meta-Analyses (PRISMA) [12]. The study protocol was created using the PICO framework, which includes population (XLP1 patients), intervention (not applicable), comparison (not applicable), and outcome (neurological manifestations and survival) (Fig. 1).

### Search strategy

We conducted a systematic and thorough search on PubMed, Scopus, Web of Science, and Embase to discover pertinent articles up to March 2025 with no restrictions on the year of publication. Keywords utilized include “X-linked lymphoproliferative,” “XLP,” “X-linked lymphoproliferative,” “XLP1,” “XLP-1,” “SH2D1A,” “neurology,” “neurologic,” “neurologic findings,” “neurologic sign*,” “neurologic symptom*,” “neurologic* manifestation*,” “central nervous system,” “CNS,” “vasculitis,” “X-linked lymphoproliferative,” “hemophagocytic lymphohistiocytosis,” “HLH,” “cerebral,” and “lymphoma” (Supplementary Table 1).

### Study selection and eligibility criteria

Our search strategy focused on English-language publications examining neurological manifestations in individuals with a genetic diagnosis of XLP1, published till March 2025. We included relevant cohort studies, letters to the editor, case reports, case series, and observational studies. Conversely, we excluded review articles, works with restricted access, posters, oral presentations, irrelevant studies, and conference abstracts.

Neurological symptoms were confirmed as XLP1-related through histopathological evidence, radiological findings consistent with XLP1-related CNS involvement, or exclusion of alternative neurological conditions. Three independent researchers conducted the screening process by examining both abstracts and full texts. All inconsistencies that emerged were addressed through consensus discussions and meetings.

### Data extraction

Data extraction encompassed the collection of the following information: the first author, year of publication, genetic testing results, age at diagnosis of XLP1, age at initial presentation, age at onset of neurological manifestations, past medical history, specifics of neurological manifestations, presence of encephalitis, vasculitis, and chorioretinitis, occurrence of lymphoma (either central nervous system or extra-CNS), systemic manifestations, HLH, hypogammaglobulinemia, and aplastic anemia (AA).

For neuroimaging data extraction, we systematically reviewed all available imaging reports from included studies. We extracted information on anatomical locations of lesions, radiological characteristics (hemorrhage, edema, enhancement patterns), and imaging modality used. Findings were categorized according to standardized anatomical regions and pathological features. When multiple imaging studies were performed on the same patient, we extracted findings from all available time points.

Additionally, cerebrospinal fluid (CSF) analysis reports, neuropathology findings, neuroradiological assessments, EBV serology and viral load, organ involvement by EBV, therapeutic options, HSCT, and survival status and cause of death were comprehensively documented.

### Quality assessment

We utilized the Joanna Briggs Institute Critical Appraisal tools for case reports, case series, and cross-sectional studies to evaluate patient selection, comparability, and outcome assessment. Studies scoring 70% or higher, 50% to 70%, and lower than 50% were considered high, moderate, and low quality, respectively.

### Statistical analysis

Categorical and quantitative variables were represented as percentages and medians (with ranges or interquartile ranges [IQRs]), and intergroup comparisons of categorical variables were analyzed using the χ^2^ test or Fisher’s exact test. Kaplan-Meier and log-rank tests were employed for survival analysis. To enhance the robustness of the survival analysis, five patients (P1, P9, P12, P19, and P31) whose survival durations were identified as outliers based on z-score analysis (|z| > 3) and visual examination of box plots and scatter plots were excluded from the Kaplan–Meier survival and log-rank analyses. Time zero for all analyses was the date of diagnosis of XLP1. Survival time was calculated from diagnosis to the event of death or censoring at the date of last follow-up, and censoring was applied at the last reported follow-up time for each patient as documented in their pertinent source studies, as there was no conventional loss to follow-up, since the data were derived from published literature. Additionally, the number of patients at risk at each time point is reported below each Kaplan–Meier curve and reflects those remaining under observation and event-free at each specified interval. A p-value of less than 0.05 was deemed statistically significant. All the analyses were conducted utilizing R version 4.4.2.

### Case report

A five-year-old Iranian boy exhibited diminished consciousness and generalized tonic-clonic seizures. In the past month, he experienced cognitive decline and manifested truncal ataxia. The analysis of his CSF showed markedly raised protein concentrations (1,780 mg/dl; normal range 15–45 mg/dl), increased white blood cell count (19/µL; 80% lymphocytes), elevated red blood cell count (75/µL; normal < 1), and diminished glucose levels (42 mg/dl; normal range 50–80 mg/dl). CSF cultures and polymerase chain reaction (PCR) tests for herpes simplex virus and enteroviruses yielded negative results. Magnetic resonance imaging (MRI) of the brain showed bilateral hyperintense lesions in cortical regions and the basal ganglia. A tentative diagnosis of acute disseminated encephalomyelitis (ADEM) was established, and the treatment included intravenous immunoglobulin (IVIG, 1 g/kg/day for two days) and high-dose methylprednisolone pulses (30 mg/kg/day for three days), succeeded by a tapering course of oral prednisolone (2 mg/kg/day). Acute symptoms have ameliorated, with minimal ataxia remaining at the follow-up assessments.

One year later, the patient presented with fever, weight loss, and abdominal pain, resulting in a diagnosis of Burkitt lymphoma of the descending colon. He received a distal colectomy and multi-agent chemotherapy, leading to remission. Subsequently, he experienced recurrent upper and lower respiratory infections. Immunological investigations demonstrated abnormal antibody responses and reversed CD4/CD8 T cell ratios.

**Fig. 2:**
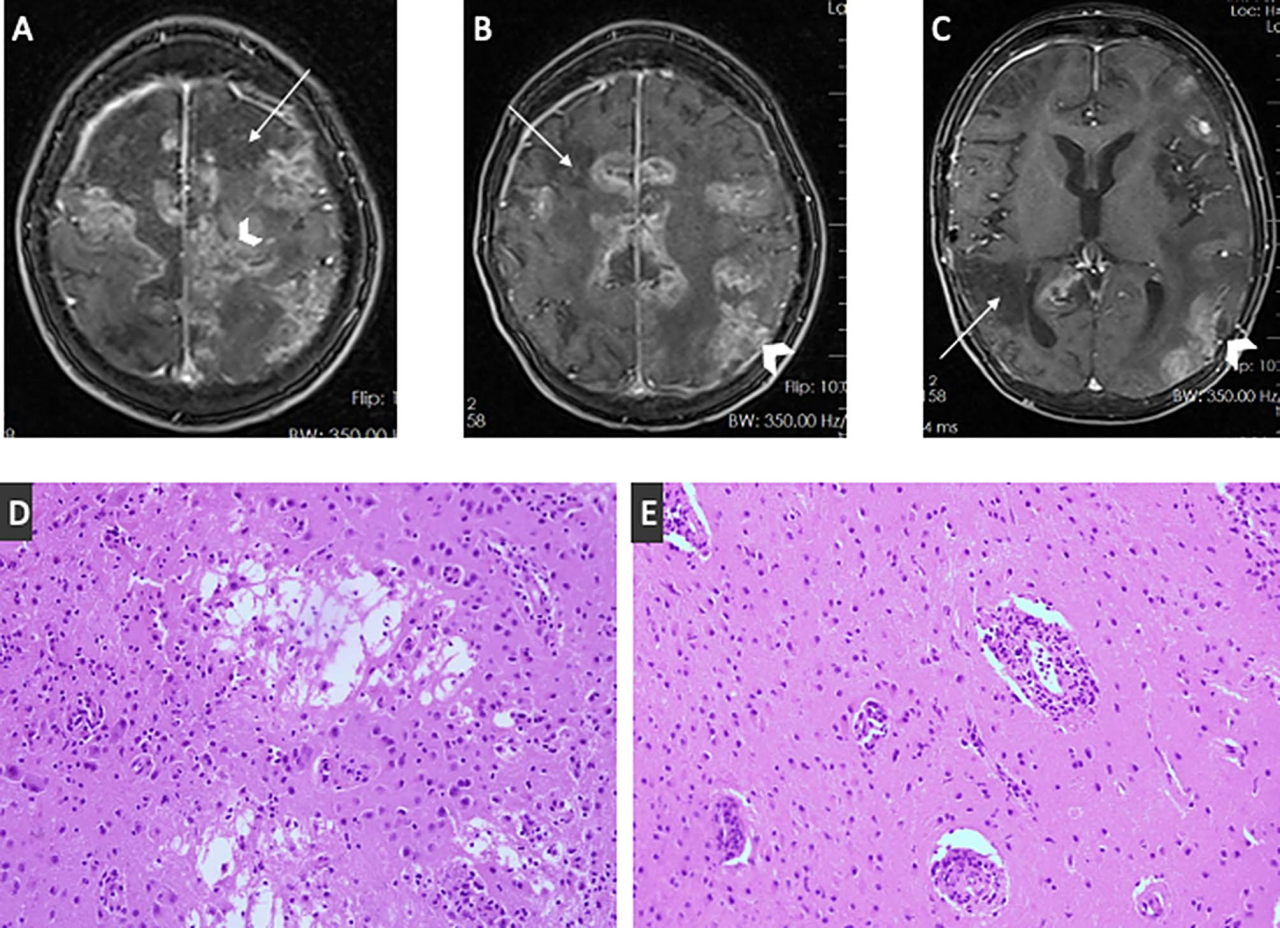
(**A-C**) contrast-enhanced axial T1-weighted fat-saturated MR images showed multiple wedge-shaped cortical-subcortical hypo-intensities in both hemispheres due to ischemic infarct (thin arrows) and patchy leptomeningeal and cortical enhancements (arrowheads). (**D**) microcystic formation and partial cavitation of brain parenchyma along with foci of chronic inflammation composed of lymphocytes and some macrophages in favor of encephalitis (HE x400). (**E**) transmural inflammation of the vessels with more than two layers of inflammatory cells mainly composed of lymphocytes in favor of vasculitis (HE x200)

At the age of ten, he exhibited fever, progressive loss of consciousness, and worsening seizures. CSF analysis revealed elevated protein levels (2,280 mg/dl), reduced glucose concentration (44 mg/dl), and lymphocytic pleocytosis, with viral testing yielding negative results. The MRI revealed several wedge-shaped cortical-subcortical hypo-intensities together with patchy leptomeningeal and cortical enhancements (Fig. 2 A to C).

Subsequent immunologic assessment revealed hypogammaglobulinemia and diminished antibody responses.

Methylprednisolone treatment (30 mg/kg/day for three days) was commenced. A CT-guided biopsy of a parietal lesion demonstrated chronic encephalitis and vasculitis (Fig. 2D and E), with brain tissue exhibiting a positive result for EBV DNA. The patient received anti-CD20 therapy (Rituximab, 375 mg/m^2^ weekly for four cycles) and monthly IVIG (500 mg/kg). Notwithstanding these measures, his neurological condition worsened, causing cognitive deterioration and confinement to bed, ultimately culminating in death from respiratory failure. Whole-exome sequencing identified a point mutation c.192 G > A (Trp64*) in exon 2 of *SH2D1A* (ENSG00000183918), causing a premature stop codon within the SH2 domain.

## Results

Our search yielded forty-one cases of XLP1 across thirty-eight studies, which included twenty-six case reports, seven case series, four correspondences, one observational study, and one novel case [8, 11, 13–47].

Of the 42 XLP1 patients with neurological involvement, 23 cases (54.8%) had histopathological confirmation through biopsy or autopsy demonstrating lymphocyte infiltration or other XLP1-associated CNS pathology. The remaining patients were diagnosed based on characteristic radiological findings consistent with XLP1-related neurological diseases and systematic exclusion of alternative neurological disorders.

In the quality assessment, all case reports were of high quality, and one cross-sectional study scored moderate. Moreover, five and three of the eight case series studies were of high and moderate quality, respectively (Supplementary Tables 2 and 3).

The clinical findings and paraclinical studies of the patients are summarized in Supplementary Table 4. The median ages (interquartile range, IQR) at initial presentation, neurologic presentation, diagnosis, and last follow-up were 4 years (2–6.25), 5 years (3–14.5), 5.37 years (4–18), and 5.37 years (4–19), respectively.

The majority of patients (38.1%; 16 out of 42) displayed low levels of immunoglobulin and were diagnosed with either agammaglobulinemia or hypogammaglobulinemia.

The neurologic involvement was in different settings.

Sixteen (38.1%) patients met diagnostic criteria for HLH. Notably, patient P41 presented with isolated CNS HLH.

Vasculitis was observed in 15 patients, with distinct patterns of presentation. Twelve (28.6%) patients had primary CNS vasculitis, while three (7.1%) patients (P30, P38, and P39) had CNS involvement secondary to systemic vasculitis.

CNS lymphoma was diagnosed in eight (19%) patients with varied presentations. 6 (14.3%) patients developed primary CNS lymphoma (including five large B-cell lymphomas and one T-cell lymphoma), while patient P4 developed post-transplant lymphoproliferative disorder (PTLD) following liver transplantation. 2 (4.8%) patients had CNS involvement as part of disseminated lymphoma: one with Hodgkin’s lymphoma (P16) and one with large B-cell lymphoma (P20). Additionally, five (11.9%) patients had a history of extra-CNS lymphomas, including three Burkitt-type lymphomas and one Hodgkin’s lymphoma. One patient (P6) developed adrenal ganglioneuroblastoma. Three patients (P23, P36, and P42) developed CNS vasculitis 6 to 48 months after diagnosis of Burkitt’s lymphoma. Finally, two patients (P37, P40) were diagnosed with limbic encephalitis.

### Neurological presentations

**Fig. 3:**
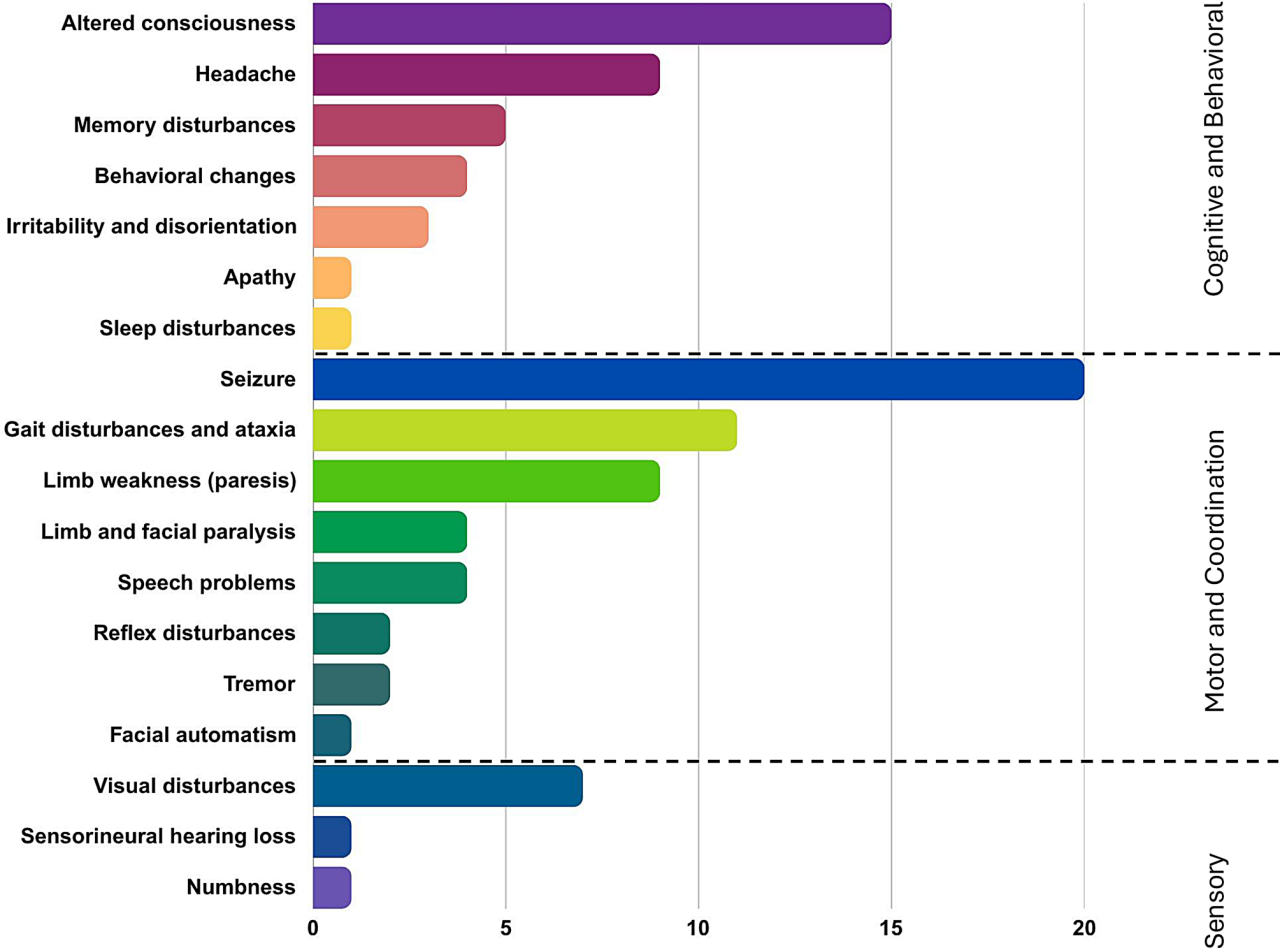
Distribution of neurological presentation frequencies

Figure 3 summarizes the frequency of neurological presentations. Among the cohort of forty-two patients, seizures emerged as the most prevalent neurological symptom, affecting approximately 47.6% (20/42) of individuals. Altered consciousness (lethargy and drowsiness) was the second most common CNS manifestation, reported in 35.7% (15/42) of patients. Notably, one patient (2.4%) experienced sleep disturbances attributed to disruptions in sleep-wake regulation.

Additional neurological symptoms included headache, reported by 21.4% (9/42) of patients.

Memory issues were present in 11.9% (5/42) of patients, with the majority (4/42) experiencing short-term memory difficulties. Behavioral changes were reported in 9.5% (4/42) of patients, all of whom were diagnosed with encephalitis. Additional symptoms included irritability and disorientation in 7.1% (3/42), while apathy was observed in 2.4% (1/42).

In terms of speech skills, 7.1% (3/42) experienced dysarthria, and dysgraphia, dyslexia, and dyspraxia were reported in 2.4% (1/42). Facial automatism was noted in one patient (2.4%, 95% CI: 0.0006–0.1256).

Motor dysfunctions included limb weakness in 21.4% (9/42) of patients and gait disturbances in 16.7% (7/42), with 9.5% (4/42) presenting with ataxia. Paralysis was found in 9.5% (4/42) of patients, including facial paralysis and quadriplegia in 4.8% (2/42), along with upper extremity paralysis in 1 (2.4%). Numbness was reported in one patient (2.4%, 1/42), and two individuals experienced reflex disturbances and tremors (4.8%).

Visual disturbances were also prevalent, affecting 16.7% (7/42) of patients, with blurred vision being the most common presentation (7.1%, 3/42). Less frequently, patients reported diplopia (4.8%, 2/42) and abnormalities in pupil examination (2.4%, 1/42). Nystagmus was observed in one patient (2.4%). Additionally, sensorineural hearing loss was reported in one patient (2.4%, 1/42).

### Neuroimaging

**Fig. 4:**
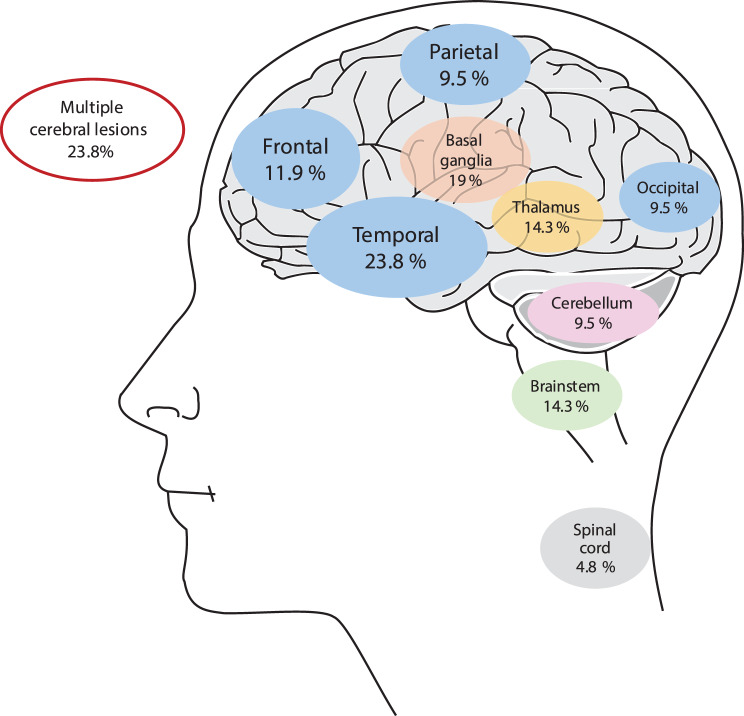
Distribution of central nervous system region involvement frequencies

The temporal lobe was the most commonly involved brain region (23.8%, 10/42), followed by the basal ganglia and related structures (19%, 8/42). The second most commonly involved cortical region was frontal, as seen in 11.9% (5/42) of patients, and the parietal and occipital lobes were involved in 9.5% (4/42), respectively. Multiple cerebral lesions were reported in 23.8% (10/42). The thalamus (14.3%, 6/42), brain stem (14.3%, 6/42), cerebellum (9.5%, 4/42), supra- and infratentorial (4.8%, 2/42), spinal cord (4.8%, 2/42), periventricular white matter (4.8%, 2/42), and corpus callosum (2.4%, 1/42) were involved (Fig. 4).

Additionally, the most commonly reported pathologies were hemorrhage (21.4%, 9/42), and edema was reported in six (14.3%) patients, which was significantly noted in patients with frontal involvement (P value 0.024). Among the less frequently reported pathologies, infarction was reported in 4.8% (2/42). Aneurysm and meningoencephalitis each occurred in 2.4% (1/42), and atrophy, demyelination, and vasculitis were reported in 4.8% (2/42).

### Cerebrospinal fluid analysis

CSF analysis reports of 45.2% (19/42) were available. Protein and white blood cells (WBCs) were elevated in 79% (15/19) and 68.5% (13/19), respectively. CSF glucose was low in 15.7% (3/19). Red blood cells were documented in the CSF in one individual (P46).

### EBV presence

The majority of patients (54.8%, 23/42) tested positive for EBV DNA. Specifically, 51.2% (21/41) exhibited positive EBV in blood, while 60% (6/10) had positive EBV in CSF. Tissue-positive EBV infections were identified in 25.8% (8/31) of the cases. Notably, four patients had tissue-positive EBV without any detectable EBV in their blood. The median EBV viral load across eight studies was reported to be 67,184 copies/mL (IQR: 13725–375,000). Also, four studies provided data on the EBV load in CSF, which revealed a median viral load of 62,700 copies/mL (IQR: 3,154.75–66,028,250).

### Genetic findings

**Fig. 5:**
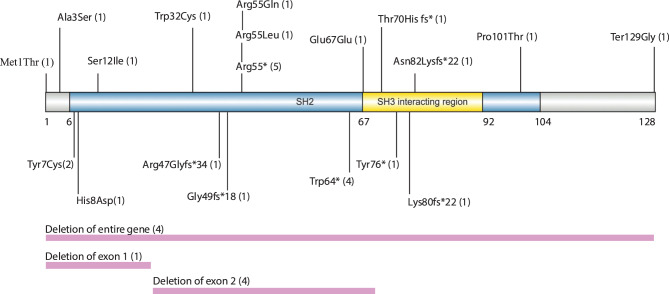
Schematic representation of the SH2D1A protein showing the reported mutations

Genetic analysis of thirty-six patients revealed twenty-two distinct *SH2D1A* mutations: eleven missense, three nonsense, five frameshift, and three large deletions (Fig. 5, Supplementary Table 5). Genetic data were unavailable for six patients: five were reported to have an unspecified *SH2D1A* mutation, and one showed absent SAP protein expression on immunoblotting. Mutations were distributed throughout the protein, with the highest concentration in the SH2 domain’s β-sheet regions essential for protein interactions. Two mutation hot spots emerged: Arg55 (seven patients) and Trp64 (four patients), both located in critical structural elements that maintain protein stability and function. Large deletions caused complete protein loss, while missense mutations in conserved residues produced varying functional impairment. These mutation types and hotspots, particularly the Arg55 and Trp64 variants, are not unique to this cohort and have been previously reported in other XLP1 patients who did not present with neurological symptoms during childhood. However, these patients may develop neurological symptoms with age.

### Systemic therapy

Corticosteroids were the most frequently administered medications, used in 69% (29/42). Among these, prednisolone was prescribed in 16.7% (7/42) of cases, methylprednisolone in 19% (8/42), and dexamethasone in 26.2% (11/42).

IVIG was the second most commonly utilized treatment, administered to 38.1% (16/42) of patients. This was followed by cyclophosphamide, used in 31.9% (15/47) of cases, and rituximab, which was administered in 33.3% (14/42). Etoposide and methotrexate were also utilized, either as standalone therapies or as part of the HLH protocol, in 33.3% (14/42) and 28.6% (12/42) of the patients, respectively. Antineoplastic agents were used in 19% of cases (8/42), while radiotherapy was employed in 9.5% (4/42).

The remaining immunosuppressive agents included cyclosporine in 16.7% (7/42), mycophenolate mofetil in 9.5% (4/42), tacrolimus in 4.8% (2/42), and azathioprine in 2.4% (1/42). Antiviral drugs, including acyclovir and ganciclovir, were used in 19% (8/42) of cases. Other treatments included antiepileptic agents in 4.8% (2/42), mannitol in 4.8% (2/42), and plasma exchange in one.

### HSCT and survival

**Fig. 6:**
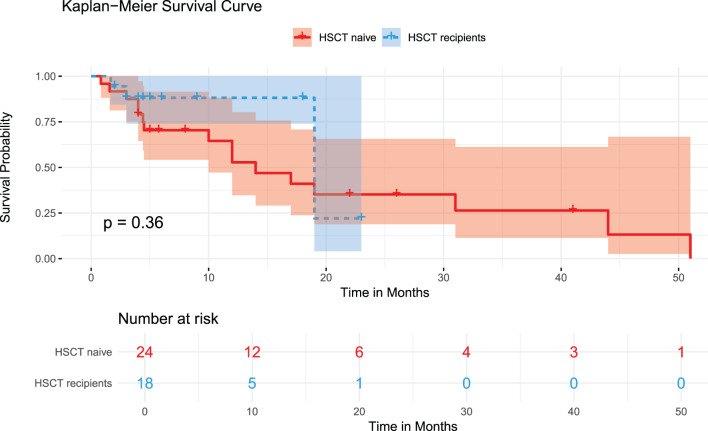
Kaplan-meier survival analysis curve (primary analysis)

Among forty-two XLP1 patients, hematopoietic stem cell transplantation (HSCT) was performed in 42.9% (18/42) of cases. Overall mortality was 52.4% (22/42), with a median age at death of 11 years (IQR: 3.75–19) and median survival time of 5.35 years (IQR: 4–19) from initial neurological symptom onset. Disease progression was rapid, with 21 patients (50%) dying within five years of symptom onset and 9 patients (21.4%) dying before age 10 years. The Kaplan–Meier analysis revealed no statistically significant difference in overall survival between HSCT recipients and HSCT-naïve patients (P = 0.36, Fig. 6). To explore the influence of extreme survival times, we conducted a sensitivity analysis excluding statistical outliers (|z| > 3). Under this condition, HSCT recipients showed higher survival than non-recipients (72.2% vs. 29.2% at 25 months; P = 0.013, Supplementary Figure 1). The survival curves diverged early in the follow-up period and continued to separate over time, with HSCT patients showing sustained therapeutic benefit.

## Discussion

Since its description in 1975, XLP1 has been acknowledged as a lethal IEI marked by uncontrolled EBV infection in males. Early neurologic findings were confined to autopsy results, revealing lymphoid infiltration in two patients from the original family who had no neurological symptoms throughout their lifetime [1, 48].

Reported XLP1 cohort studies have primarily concentrated on systemic manifestations. Among 182 patients across five studies, hemophagocytic lymphohistiocytosis/fulminant infectious mononucleosis (HLH/FIM) and dysgammaglobulinemia were the most prevalent conditions (59.3% each), followed by lymphoproliferative disease (27%) [5, 49–52]. Neurological manifestations were observed in merely 7.1% of patients, indicating under-recognition. Recent case reports have progressively expanded our understanding of XLP1‘s neurologic spectrum.

This systematic review offers a thorough study of neurological manifestations in XLP1, revealing a clinically heterogeneous yet consistently severe range of CNS involvement characterized by high morbidity and mortality. Our analysis indicates that neurological problems are prevalent but may serve as the early manifestation of XLP1, often preceding systemic features [29, 39]. The quartet of CNS pathologies—HLH, vasculitis, lymphoma, and limbic encephalitis—demonstrates the intricate immune dysregulation, with each condition adding to the disease burden and diagnostic complexity.

Although 38.1% of patients met the criteria for HLH, aligning with the reported incidence of 30–55% [5, 51–53], CNS involvement surpassed HLH-associated presentations.

Likewise, CNS vasculitis, which affected approximately one-third of the cohort, frequently manifested as a primary CNS condition and occasionally in the absence of detectable EBV infection, as previously reported [29, 40]. This indicates that the intrinsic immunological dysregulation of SH2D1A deficiency is sufficient to initiate vascular inflammation, irrespective of a particular viral trigger. To assess whether SH2D1A deficiency intrinsically drives vasculitis, we propose specific testable investigations. These include comparative immunohistochemistry and transcriptomic profiling of EBV-negative versus EBV-positive CNS lesions to identify distinct inflammatory signatures, ex vivo SAP-deficient models to examine baseline immune activation, and systematic comparing of CSF inflammatory profiles between EBV-status groups.

CNS lymphoma, whether primary or secondary, remains a significant contributor to morbidity and mortality, aligning with the established predisposition to B-cell malignancies in XLP1 [4, 5].

In our sample and the literature we reviewed, several XLP1 patients developed CNS vasculitis months after Burkitt’s lymphoma; while this pattern is noteworthy, current evidence is insufficient to conclude disease-specific exclusivity [33, 46, 54].

Two individuals had limbic encephalitis; one had anti-AMPAR autoantibodies, suggesting that B-cell-targeted therapy could be advantageous for patients with this presentation [11, 42].

The varied neurological manifestations of XLP1 present considerable diagnostic challenges. In our group, the predominant initial symptoms—seizures, altered consciousness, and headaches—may resemble viral encephalitis or autoimmune conditions.

This clinical heterogeneity reflects the documented underlying pathologies. Our review found a tendency for inflammatory and hemorrhagic lesions in the temporal lobes and basal ganglia, correlating with common symptoms such as seizures, memory impairments, motor dysfunction, and altered consciousness. Despite being non-specific, CSF results of elevated protein and pleocytosis suggest active neuroinflammation and warrant XLP1 evaluation in at-risk males presenting with unexplained encephalitis.

The role of EBV in CNS disease is complex. EBV DNA was identified in more than fifty percent of the patients; however, a significant number had CNS involvement without systemic viremia, and some had EBV found solely in CSF or CNS tissue, including two tissue-positive, blood-negative patients. This organ-positive/blood-negative pattern reveals a critical diagnostic blind spot, where reliance on peripheral blood assays may obscure localized EBV-driven pathology and underscores the significance of site-specific viral testing, as blood-based EBV testing alone may prove inadequate and potentially misleading [48].

The differential diagnosis for neurologic involvement in XLP1 is extensive, as its presentations overlap with infectious, autoimmune, and other genetic diseases. Distinguishing XLP1-related neuroinflammation from CNS infections (especially EBV) and post-infectious disorders such as ADEM (as observed in P42) poses significant challenges. We must also consider and exclude other IEIs, including primary HLH syndromes (e.g., familial HLH subtypes and XIAP deficiency), type 1 interferonopathies, and genetic vasculopathies such as DADA2 (Deficiency of Adenosine Deaminase 2) [55, 56].

The genetic analysis revealed no definitive genotype-phenotype correlation regarding neurological involvement; however, some mutation hot spots were more frequent in the series. Individuals with XLP1 carrying the Arg55 mutation may be more prone to developing neurological symptoms with age compared to other mutations, though the underlying mechanisms need further study. The lack of universal genetic predictors for CNS involvement highlights the necessity for diligent neurological monitoring in all XLP1 patients, irrespective of their particular mutation.

Our findings underscore the significant limitations of conventional therapy in the management of XLP1. Notwithstanding the prevalent application of immunomodulatory and cytotoxic therapies, the disease continues to be highly lethal, with a median survival of merely five years from the onset of neurological symptoms. While HSCT has been considered potentially beneficial, our aggregated case-based analysis did not show a statistically significant survival difference between HSCT recipients and non-recipients in the primary analysis. A sensitivity analysis excluding statistical outliers suggested a possible survival advantage with HSCT, but this finding is exploratory and highly sensitive to data handling choices. Accordingly, these results should be regarded as hypothesis-generating rather than definitive.

Finally, we would like to recognize the limitations of our study. The scarcity of reports regarding the CNS characteristics of this population, along with the lack of definitive randomized controlled trials and observational studies, may have influenced the results of this review, as our conclusions are exclusively derived from case reports, case series, correspondences, and one observational study. The mortality rates among persons with neurologic manifestations summarized in this study warrant additional assessment of therapeutic alternatives for this population.

## Conclusions

This systematic review highlights the critical need for increased clinical awareness of neurological manifestations in XLP1. These manifestations are not only varied and perhaps the early indicators of disease, but also closely linked with adverse outcomes. Timely diagnosis and early referral for HSCT are crucial for enhancing survival rates. Given that neurologic manifestations may occur prior to systemic signs or resemble other inflammatory or infectious CNS disorders, clinicians should remain vigilant for XLP1 in male patients exhibiting unexplained neuroinflammation or lymphoma. Future efforts should focus on establishing diagnostic criteria and early HSCT protocols to mitigate the considerable morbidity and mortality linked to CNS involvement in XLP1.

## Electronic supplementary material

Below is the link to the electronic supplementary material.

Supplementary Material 1: Kaplan-Meier survival analysis curve (Sensitivity analysis after exclusion of outliers)

Supplementary Material 2

Supplementary Material 3

Supplementary Material 4

Supplementary Material 5

## Data Availability

The genetic data from the patients described in this study have been submitted to the Global Variome shared LOVD database and are publicly accessible at: https://databases.lovd.nl/shared/individuals/00466061

## Abbreviations

AA: Aplastic anemia
ADEM: Acute disseminated encephalomyelitis
Anti-AMPAR: Anti-Alpha-Amino-3-Hydroxy-5-Methyl-4-Isoxazolepropionic Acid
CNS: Central nervous system
CT: Computed tomograph
EBV: Epstein-Barr virus
FIM: Fulminant infectious mononucleosis
HLH: Hemophagocytic lymphohistiocytosis
HSCT: Hematopoietic stem cell transplantation
IEI: Inborn error of immunity
IVIG: Intravenous immunoglobulin
IQRs: Interquartile ranges
MRI: Magnetic resonance imaging
PCR: Polymerase chain reaction
PTLD: Post-transplant lymphoproliferative disorder
PRISMA: Preferred Reporting Items for Systematic Reviews and Meta-Analyse
SAP: Signaling lymphocyte activation molecule (SLAM)-associated protein
XLP1: X-linked lymphoproliferative syndrome type 1
