# Supplementary figures and images for: The neurologic face of X-linked lymphoproliferative syndrome type 1: a systematic review
Source: Orphanet J Rare Dis. 2025 Oct 21;20:528. doi: 10.1186/s13023-025-04057-9 (PMC12541938; doi:10.1186/s13023-025-04057-9)

## Kaplan-Meier Survival Curve

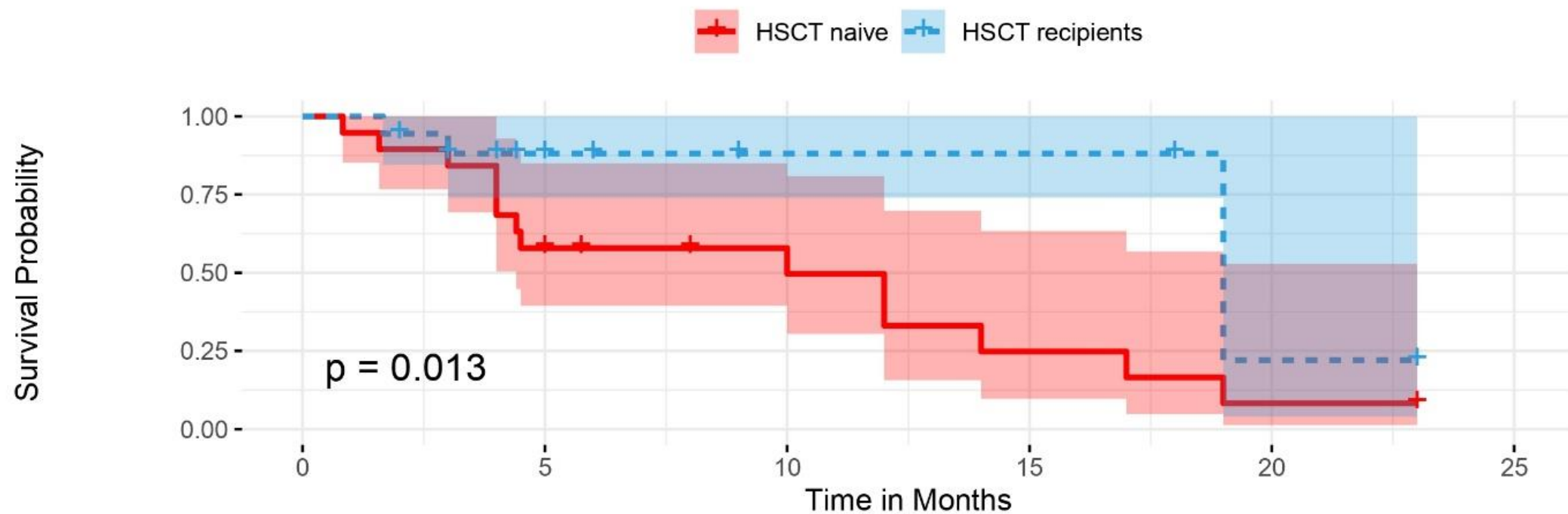

## Number at risk

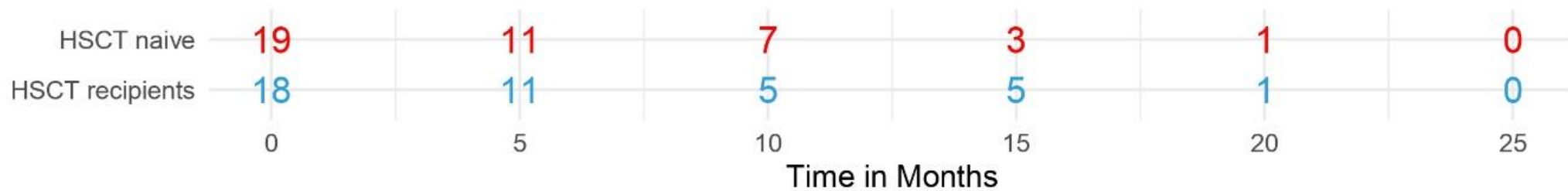

Supplement: Supplementary file 1 — Supplementary Material 1: Kaplan-Meier survival analysis curve (Sensitivity analysis after exclusion of outliers) [file 13023_2025_4057_MOESM1_ESM.pdf]
