# Supplementary material for: The neurologic face of X-linked lymphoproliferative syndrome type 1: a systematic review
Source: Orphanet J Rare Dis. 2025 Oct 21;20:528. doi: 10.1186/s13023-025-04057-9 (PMC12541938; doi:10.1186/s13023-025-04057-9)
Supplement: Supplementary file 2 — Supplementary Material 2 [file 13023_2025_4057_MOESM2_ESM.docx]

**Supplementary Table 1.** Detailed search strategy in databases

| **Search query** | | **Results** |
| --- | --- | --- |
| **PubMed** | | |
| **#1** | (((((((((((("Neurology"[Mesh]) OR ("Central Nervous System"[Mesh])) OR ("neurologic"[Text Word])) OR ("Neurologic Manifestations"[Mesh])) OR ("Neurologic Finding*"[Text Word])) OR ("Neurologic Sign*"[Text Word])) OR ("Neurologic Symptom*"[Text Word])) OR ("Neurologic* Manifestation*"[Text Word]))) OR ("Cerebrum"[Mesh])) OR ("Brain"[Mesh]) ) AND ((((((((((("X-Linked Lymphoproliferative Disorder"[Text Word])) OR ("X-Linked Lymphoproliferative "[Text Word])) OR ("XLP"[Text Word])) OR ("XLP1"[Text Word]])) OR ("X-Link Lymphoproliferative"[Text Word])) OR ("X Linked Lymphoproliferative"[Text Word])) OR ("XLinked Lymphoproliferative "[Text Word])) OR ("X Link Lymphoproliferative "[Text Word])) OR (X-linked lymphoproliferative syndrome[Text Word])) OR ("SH2D1A"[Text Word])) | **17** |
| **Embase** | | |
| **#2** | **('neurology'/exp OR neurology OR 'central nervous system'/exp OR 'central nervous system' OR neurologic OR 'neurologic manifestations'/exp OR 'neurologic manifestations' OR 'neurologic finding*' OR 'neurologic sign*' OR 'neurologic symptom*' OR 'neurologic* manifestation*' OR 'cerebrum'/exp OR cerebrum OR 'brain'/exp OR brain) AND ('x-linked lymphoproliferative disorder'/exp OR 'x-linked lymphoproliferative disorder' OR 'x-linked lymphoproliferative' OR xlp OR xlp1 OR 'x-link lymphoproliferative' OR 'x linked lymphoproliferative' OR 'xlinked lymphoproliferative' OR 'x link lymphoproliferative' OR 'x-linked lymphoproliferative syndrome'/exp OR 'x-linked lymphoproliferative syndrome' OR sh2d1a)** | 254 |
| **Web of science** | | |
| **#3** | **((((((((((((ALL=Neurology) OR (ALL="Central Nervous System")) OR (ALL=neurologic)) OR (ALL="Neurologic Manifestations")) OR (ALL="Neurologic Finding*")) OR (ALL="Neurologic Sign*")) OR (ALL="Neurologic Symptom*")) OR (ALL="Neurologic* Manifestation*"))) OR (ALL=Cerebrum)) OR (ALL=Brain)) AND (((((((((((ALL="X-Linked Lymphoproliferative Disorder")) OR (ALL="X-Linked Lymphoproliferative")) OR (ALL=XLP)) OR (ALL=XLP1])) OR (ALL="X-Link Lymphoproliferative")) OR (ALL="X Linked Lymphoproliferative")) OR (ALL="XLinked Lymphoproliferative")) OR (ALL="X Link Lymphoproliferative")) OR (ALL="X-linked lymphoproliferative syndrome")) OR (ALL=SH2D1A)))** | **29** |
| **Scopus** | | |
| **#4** | **( ( ( ( ( ( ( ( ( ( ( ( INDEXTERMS ( Neurology ) ) OR ( INDEXTERMS ( "Central Nervous System" ) ) ) OR ( TITLE-ABS-KEY ( neurologic ) ) ) OR ( INDEXTERMS ( "Neurologic Manifestations" ) ) ) OR ( TITLE-ABS-KEY ( "Neurologic Finding*" ) ) ) OR ( TITLE-ABS-KEY ( "Neurologic Sign*" ) ) ) OR ( TITLE-ABS-KEY ( "Neurologic Symptom*" ) ) ) OR ( TITLE-ABS-KEY ( "Neurologic* Manifestation*" ) ) ) ) OR ( INDEXTERMS ( Cerebrum ) ) ) OR ( INDEXTERMS ( Brain ) ) ) AND ( ( ( ( ( ( ( ( ( ( ( TITLE-ABS-KEY ( "X-Linked Lymphoproliferative Disorder" ) ) ) OR ( TITLE-ABS-KEY ( "X-Linked Lymphoproliferative" ) ) ) OR ( TITLE-ABS-KEY ( XLP ) ) ) OR ( TITLE-ABS-KEY ( XLP1] ) ) ) OR ( TITLE-ABS-KEY ( "X-Link Lymphoproliferative" ) ) ) OR ( TITLE-ABS-KEY ( "X Linked Lymphoproliferative" ) ) ) OR ( TITLE-ABS-KEY ( "XLinked Lymphoproliferative" ) ) ) OR ( TITLE-ABS-KEY ( "X Link Lymphoproliferative" ) ) ) OR ( TITLE-ABS-KEY ( "X-linked lymphoproliferative syndrome" ) ) ) OR ( TITLE-ABS-KEY ( SH2D1A ) ) ) )** | **60** |
