# Supplementary material for: The neurologic face of X-linked lymphoproliferative syndrome type 1: a systematic review
Source: Orphanet J Rare Dis. 2025 Oct 21;20:528. doi: 10.1186/s13023-025-04057-9 (PMC12541938; doi:10.1186/s13023-025-04057-9)
Supplement: Supplementary file 3 — Supplementary Material 3 [file 13023_2025_4057_MOESM3_ESM.docx]

**Supplementary Table 2:** Quality assessment of case reports and cross-sectional studies using JBI critical appraisal checklist for case reports and cross-sectional studies

| **Study** | **D1** | **D2** | **D3** | **D4** | **D5** | **D6** | **D7** | **D8** | **Score** | **Overall quality score** |
| --- | --- | --- | --- | --- | --- | --- | --- | --- | --- | --- |
| **Blackburn 2019** | Y | Y | Y | Y | Y | Y | Y | Y | 100% | High |
| **Børresen 2019** | Y | Y | Y | Y | Y | Y | Y | Y | 100% | High |
| **Chartier 2021** | Y | Y | Y | Y | Y | Y | Y | Y | 100% | High |
| **Ghosh 2022** | Y | Y | Y | Y | Y | Y | Y | Y | 100% | High |
| **Goodyer 2013** | Y | Y | Y | Y | Y | Y | Y | Y | 100% | High |
| **Gray 2015** | Y | Y | Y | Y | Y | Y | Y | Y | 100% | High |
| **Hervier 2010** | Y | Y | Y | Y | Y | Y | Y | Y | 100% | High |
| **Hügle 2007** | Y | Y | Y | Y | Y | Y | Y | Y | 100% | High |
| **Jiang 2020** | Y | Y | Y | Y | Y | Y | Y | Y | 100% | High |
| **Karasawa 2021** | Y | Y | Y | Y | Y | Y | Y | Y | 100% | High |
| **Korah-sedwig 2018** | Y | Y | Y | Y | Y | Y | Y | Y | 100% | High |
| **Kusano 2019** | Y | Y | Y | Y | Y | Y | Y | Y | 100% | High |
| **Kwon 2022** | Y | Y | Y | Y | N | Y | Y | Y | 87.5% | High |
| **Li 2024** | Y | Y | Y | Y | Y | Y | Y | Y | 100% | High |
| **Liu 2015** | Y | Y | Y | Y | N | Y | Y | Y | 87.5% | High |
| **Mukai 2023** | Y | Y | Y | Y | N | Y | Y | Y | 87.5% | High |
| **Nallasamy 2011** | Y | Y | Y | Y | N | Y | Y | Y | 87.5% | High |
| **Neves 2019** | Y | Y | Y | Y | N | Y | Y | Y | 87.5% | High |
| **Ortega 2013** | Y | Y | Y | Y | Y | Y | Y | Y | 100% | High |
| **Sankararaman 2014** | Y | Y | Y | Y | N | Y | N | Y | 75% | High |
| **Steininger 2021** | Y | Y | Y | Y | N | Y | Y | Y | 87.5% | High |
| **Voeten 2014** | Y | Y | Y | Y | Y | Y | Y | Y | 100% | High |
| **Weeks 2006** | Y | Y | Y | Y | N | Y | Y | Y | 87.5% | High |
| **Wu 2022** | Y | Y | Y | Y | N | Y | Y | Y | 87.5% | High |
| **Zhu 2013** | Y | Y | Y | Y | Y | Y | Y | Y | 100% | High |
| **Ochiai 2022** | Y | Y | Y | Y | N | Y | Y | Y | 87.5% | High |
| **Kanegane 2005** | Y | Y | Y | Y | N | Y | Y | Y | 87.5% | High |
| **Dutz 2001** | Y | Y | Y | Y | N | Y | Y | Y | 87.5% | High |
| **Verhelst 2007** | Y | Y | Y | Y | N | Y | Y | Y | 87.5% | High |
| **Trottestam 2009^*^** | Y | Y | Y | Y | N | N | Y | N | 62.5% | Moderate |

_* This study was evaluated using the JBI critical appraisal checklist for analytical cross-sectional studies_

**Supplementary Table 3:** Quality assessment of case series studies using JBI critical appraisal checklist for case series studies

| **Study** | **D1** | **D2** | **D3** | **D4** | **D5** | **D6** | **D7** | **D8** | **D9** | **D10** | **Score** | **Overall quality assessment** |
| --- | --- | --- | --- | --- | --- | --- | --- | --- | --- | --- | --- | --- |
| **Escaron 2022** | Y | Y | Y | N | N | Y | Y | Y | Y | N/A^*^ | 77.7% | High |
| **Mejstríková 2012** | N | Y | Y | N | N | Y | Y | Y | Y | Y | 70% | High |
| **Nademi 2019** | N | Y | Y | N | N | Y | Y | Y | Y | N/A | 66.6% | Moderate |
| **Parida 2022** | Y | Y | Y | N | N | Y | Y | Y | Y | Y | 80% | High |
| **Sperl 2012** | Y | Y | Y | N | N | Y | Y | Y | Y | N/A | 77.7% | High |
| **Talaat 2009** | Y | Y | Y | N | N | N | Y | Y | N | N/A | 55.5% | Moderate |
| **Bohne 2013** | N | Y | Y | N | N | Y | Y | Y | N | N/A | 55.5% | Moderate |
| **Sheth 2019** | Y | Y | Y | N | N | Y | Y | Y | Y | N/A | 77.7 | High |

_*N/A = Not applicable_
