# Supplementary material for: The neurologic face of X-linked lymphoproliferative syndrome type 1: a systematic review
Source: Orphanet J Rare Dis. 2025 Oct 21;20:528. doi: 10.1186/s13023-025-04057-9 (PMC12541938; doi:10.1186/s13023-025-04057-9)
Supplement: Supplementary file 4 — Supplementary Material 4 [file 13023_2025_4057_MOESM4_ESM.docx]

**Supplementary Table 4:** Detailed clinical and paraclinical findings of the patients

| **Pn** | **Author,**  **Country,**  **year** | **Age at XLP diagnosis** | **Age at initial presentation** | **Age at neurological symptom onset** | **Neurological symptoms summary** | **Key clinical features** | **CSF analysis summary** | **Neuropathology** | **Neuroradiology** | **Systemic and Hematologic Manifestations** | **EBV* status** | **Treatment and HSCT* status** | **Death** |
| --- | --- | --- | --- | --- | --- | --- | --- | --- | --- | --- | --- | --- | --- |
| P1 | Blackburn, P. R., et al., USA (2019) (1)  Case report | 49 | 49 | 49 | Numbness, motor weakness, functional impairment requiring walker, dysarthria | Encephalitis,  vasculitis | normal cerebrospinal fluid profile | Lymphocytic meningoencephalitis, perivasculitis, focal necrosis, and arachnoid fibrosis; **Postmortem autopsy:**  cortical infarction and hemorrhage, wallerian degeneration, gliosis, noninflammatory arteriopathic changes, vascular amyloidosis, vasculitis | **Initial MRI*:**  CNS lymphocytic vasculitis  **Follow-up MRI:** new or ongoing inflammatory activity |  | Negative serology and tissue EBV | CS*, CYC* | At 51 years old |
| P2 | Bohne, S., et al., Germany (2013) (2)  Case series | 19 | 19 | 19 | 0 | 0 |  | Hemorrhage |  | Systemic manifestations, HLH*, lymphoproliferation | Positive EBV serology | CS, antipyretics, antimicrobials, etoposide, CYC, RTX* | At 19 years old |
| P3 | Børresen, M. L., et al., Denmark (2019) (3)  Case report | 2 | 2 | 2 | Seizure, lethargy, apathy, motor agitation, sleep difficulties | encephalitis | Elevate WBCs*, protein |  | Signal changes in pons and thalamus | Systemic manifestations, HLH | Positive EBV serology. Negative EBV in tissue, 113000, 84000, and 6500 copies/ml in CSF and blood | levetiracetam, sodium oxabate, lorazepam, clonidin, RTX, etoposide, MTX*, CS, HSCT |  |
| P4 | Chartier, M.-E., et al., UK (2021) (4)  Case report | 5 | 3 | 5 | Tonic-clonic seizures six months after liver transplantation | CNS lymphoma |  | EBV-driven B-cell lymphoid proliferation | Focal lesions in the temporal regions | Systemic manifestations, hypogammaglobinemia, aplastic anemia | Positive serology, plasma viral load of 4,100,000 copies/mL, negative EBV in tissue | Antimicrobials, CS, rATG*, MMF*, AZA*, RTX, MTX, cytarabine, etoposide, FLU*, treosulfan, mAb*, CYC, HSCT |  |
| P5 | Escaron, C., et al., UK (2022) (5)  Case series | 4 | 4 | 4 | Drowsiness, irritability, generalized tonic-clonic seizures |  |  |  |  | Systemic manifestations, HLH | Positive serology | Etoposide, CS, Cyclosporine A, MTX | + |
| P6 | Ghosh, T., et al., USA (2022) (6)  Correspondence | 2 | 2 | 2 | febrile seizure | Ganglioneuroblastoma |  |  |  | Systemic manifestations | Positive serology, negative in tissue | IVIG*, FLU, thiotepa, melphalan, rATG, tacrolimus, MMF,  HSCT |  |
| P7 | Goodyer, M., et al., Ireland (2013) (7)  Correspondence | 5 | 5 | 5 | Left-sided weakness, gait disturbance | Cerebral T-cell non-Hodgkin’s lymphoma |  | CNS lymphoma | Multifocal cerebral lesions | HLH | Positive serology, negative in tissue | RTX, busulphan/CYC, MTX, ciclosporin, HSCT |  |
| P8 | Gray, P. E., et al., Australia (2015) (8)  Case report | 7 | 5 | 9 | Left orbital headache, unilateral blurred vision, occasional emesis | Vasculitis, progressive limb movements restriction with associated soft tissue swelling, synovitis, alveolitis, pangastritis, severe bladder injury due to BK* virus, anti-GBM disease | Elevate WBCs, and protein |  | Infarction, diffuse vascular irregularity in supra and infratentorial, pseudoaneurysm | Systemic manifestations, hypogammaglobinemia, aplastic anemia, lymphoproliferation |  | HCQ*, CS, antimicrobials, aspirin, mAb, treosulphan, FLU, CYC, RTX, HSCT |  |
| P9 | Hervier, B., et al., France (2010) (9)  Case report | 41 | 41 | 41 | Febrile confusion, left facial paralysis, horizontal diplopia | B-cell cerebral lymphoma, asthenia, laryngitis, odynophagia |  | CNS lymphoma | Bilateral basal ganglia lesions in basal ganglia, edema | Systemic manifestations, agammaglobulinemia | Positive serology and tissue | MTX, radiotherapy, IVIG |  |
| P10 | Hügle, B., et al., Germany (2007) (10)Case report | 4 | 4 | 4 | Tetraplegia, medullary infarction, hydrocephalus | B-cell cerebral lymphoma | Elevate WBCs, and protein | Necrosis, edema, CNS lymphoma |  | Systemic manifestations, HLH | Positive serology and tissue | Etoposide, CS, CYC | At 4 years old |
| P11 | Sheth J., et al. , India (2019) (11)Case series | 16 months | 16 months | 19 months | Focal seizure | Encephalitis | Aseptic Meningitis |  | Meningoencephalitis | Systemic manifestations, HLH | Positive serology |  | At 19 months |
| P12 | Jiang, Y., et al., USA (2020) (12) case report | 43 | 6 | 13 | Sudden vision change, headache, right-sided weakness, seizure | Hodgkin lymphoma, recurrent strokes, seizure, adrenal insufficiency |  | Necrotizing CNS vasculitis | Acute infarction |  | Negative serology | CS, CYC, IVIG, IFN-γ* | At 44 years old |
| P13 | Karasawa, T., et al., Japan (2021) (13) Correspondence | 6 | 6 | 6 | Headache, somnolence, disorientation, left arm weakness and right arm paralysis, gait disturbance, decreased consciousness, stiff neck | Encephalitis | Elevate WBCs, and protein |  | High-intensity lesions in the left parietal lobe and meninges | Systemic manifestations, hypogammaglobinemia | Negative serology | Antimicrobials, IVIG, CS, HSCT |  |
| P14 | Korah-Sedgwick, M., et al., USA (2018) (14)case report | 5 | 2 | 5 | Acute behavioral changes | Encephalitis, recurrent sinopulmonary infections | Elevated WBCs |  | Non-enhancing foci on in frontal lobe and subcortical white matter | HLH | Positive serology, 2300 copies/ml | IVIG, antimicrobials, RTX, CS, HSCT |  |
| P15 | Kusano, N., et al., Japan (2019) (15)case report | 18 | 18 | 18 | Convulsion, decreased consciousness, quadriplegia, bilateral facial paralysis, bilateral sensorineural hearing loss, dysphagia | Cerebral lymphoma, intestinal malignant lymphoma, idiopathic arthropathy | Elevate WBCs and protein | CNS lymphoma | High-intensity lesions on in occipital lobes | Systemic manifestations, HLH | Positive serology, 9x10^4^ copies/ microgram DNA | CYC or etoposide, CS, PE*, MTX, RTX, cytarabine, temozolomide, radiotherapy, fludarabine, melphalan, cytarabine, HSCT |  |
| P16 | Kwon, W. K., et al., South Korea (2022) (16)case report | 5 | 3 | 5 | Headache, left sided motor weakness | Hodgkin's lymphoma, recurrent mycoplasma pneumonias |  |  | lymphoproliferative disorder or encephalitis associated with EBV infection | Systemic manifestations, lymphoproliferation | Positive serology and tissue, EBC copies 530 IU/ml | IVIG, CYC, vincristine, CS, procarbazine hydrochloride, doxorubicin hydrochloride, bleomycin, vinblastine sulfate, RTX |  |
| P17 | Li, B., et al., China (2024) (17) case report | 4 years and 5 months old | 4 years and 5 months old | 4 years and 5 months old | Gait disturbances, tremors, slow speech, jaw and limb tremors, decreased muscle tone in all limbs, hyperactive knee jerks, sudden vision changes, vomiting, generalized seizures, areflexia on left side | Encephalitis | Elevate WBCs, protein, and low glucose | Lymphocyte infiltration | Cerebral edema;  hemorrhage, acute necrotizing encephalitis or vasculitis | HLH | Negative serology | Phenol sulfonamide, hemocoagulase Bothrops Atrox, mannitol, antimicrobials | At 4 years old |
| P18 | Liu et al., China (2015) (18)case report | 4 | 4 | 4 | Lethargy, slurred speech, convulsion, lymphocytic choriomeningitis | Recurrent respiratory infections |  |  | Low density cerebral lesions, hemorrhage | Systemic manifestations, hypogammaglobinemia | Negative serology | Antimicrobials, γ-globulin | At 4 years old |
| P19 | Mejstríková et al., Czech Republic (2012) (19)  Case series | 26 | 3 | 3 | Focal epileptiform discharges,  ADHD, dyslexia, dysgraphia, slow speech development, seizure | 0 |  |  |  | Systemic manifestations, hypogammaglobinemia | Negative serology | Antiepileptics, CS |  |
| P20 | Mukai et al., Japan (2023) (20)  Case report | 3 | 3 | 3 | Lethargy, decreased response  to stimuli, tonic–clonic  seizure | Encephalitis, fever lasting a month | Elevate WBCs, protein, and low glucose |  | Bone-destructive lesion in right nasal cavity | Systemic manifestations, lymphoproliferation | Positive serology, 470,000 EBV copies | Cyclosporin, etoposide, γ-globulin, CS, RTX, HSCT | At 3 years old |
| P21 | Nademi et al., UK, (2019) (21)  Correspondence | 4 months | 4 months | 11 months | Generalized seizure | Generalized seizure | Elevated protein | Lymphocytic infiltration | Cerebra; and cerebellar signal abnormalities, brain stem herniation | HLH | Negative serology | Etoposide, mAb, HSCT | At 20 months |
| P22 | Nallasamy et al, USA (2011) (22)  Case report | 18 years | 18 months | 18 years | Ataxia, nystagmus | Necrotizing vasculitis, chorioretinitis, idiopathic thrombocytopenic purpura, cellulitis | Elevated protein |  | Hemorrhage | Hypogammaglobinemia | Negative serology | Antimicrobials, CS, MTX, IVIG, radiotherapy, chemotherapy, HSCT | At 19 years old |
| P23 | Neves et al., Portugal (2019) (23)  Case report | 14 years | 14 years | 14 years | Short-term memory deficit | Vasculitis, Burkitt lymphoma |  |  | Hemorrhage, edema | Hypogammaglobinemia | Negative serology | Antimicrobials, IVIG, CS, RTX, MTX, CYC, mAb | At 14 years old |
| P24 | Ortega et al., Spain (2013) (24)  Case report | 10 months | 10 months | 10 months | Generalized tonic-clonic seizure | Recurrent URIs* |  | Lymphocytic infiltration | Hemorrhage, edema | Systemic manifestations | Positive serology, 50,368 EBV copies |  | At 10 months |
| P25 | Sankararaman, USA (2014) (25)  Case report | 4.5 | 4.5 | 4.5 | 0 | 0 |  |  | Hemorrhage, neuronal loss, increased neuronal membrane turnover |  | Positive serology and tissue |  | At 4.5 years old |
| P26 | Sperl et al., Austria (2012) (26)  Case series | 3 | 3 | 3 | Brain stem herniation | Encephalitis |  |  |  |  | Positive serology |  | At 3 years old |
| P27 | Sperl et al., Austria (2012) (26)  Case series | 23 | 6 months | 6 months | Intellectual disability, progressive confusion, and facial-oral  automatisms | Meningoencephalitis, vasculitis, recurrent URIs |  |  | Unspecific subcortical  white matter alterations, cerebral atrophy | Hypogammaglobinemia | Positive serology and tissue | IVIG, CS, HSCT |  |
| P28 | Steininger et al., Austria (2021) (27)  Case report | 8 yr | 3 yr | 8 yr | Headaches, behavioral changes,  left-side hemiparesis, central diabetes insipidus, increased myotonus,  decreased consciousness, flaccid tetraparesis, areflexia | Meningoencephalitis, vasculitis, recurrent URIs, one episode of pneumonia | Elevated WBCs and protein | Immune cell infiltration and vascular involvement, fibrinoid necrosis | Necrotizing lesions, intraventricular hemorrhage, fusiform aneurysms | Systemic manifestations, hypogammaglobinemia | Negative serology | Antimicrobials, MMF, CS |  |
| P29 | Talaat et al., USA (2009) (28)  Case series | 18 yr | 18 yr | 18 yr | Headache, blurred vision | Vasculitis |  | Immune cells vascular infiltration, vascular and tissue necrosis | Inflammatory vasculitis, hemorrhage | Hypogammaglobinemia | Negative serology | CS, VIGI, MTX, RTX, mAb, etoposide, sirolimus, CYC, radiotherapy, HSCT | At 19 years old |
| P30 | Talaat et al., USA (2009) (28)  Case series | 28 | 27 | 31 | Headache | Vasculitis |  | Immune cells vascular infiltration |  | Systemic manifestations | Negative serology |  | At 31 years old |
| P31 | Trottestam et al., Sweden (2009) (29)  Cross sectional | 53 months | 53 months | 53 months | Febrile convulsion |  | Elevate WBCs and protein |  |  | HLH | Negative serology | Cyclosporine, MTX, CS, MUD* transplant, busulfan, CYC, ATG, etoposide, HSCT |  |
| P32 | Trottestam et al., Sweden (2009) (29)  Cross sectional | 69 months | 69 months | 69 months | Drowsiness |  | Elevate WBCs and protein |  | Edema | HLH | Positive serology | CS, etoposide, MTX, ciclosporin |  |
| P33 | Voeten et al., Belgium (2014) (30)  Case report | 3 | 3 | 3 | Decreased consciousness, focal epileptic seizures, dyspraxia, ataxia, disturbed orientation | Recurrent URIs | Xanthochromia, elevated protein, RBC count at 5 cells/μL |  | Cortical and subcortical high intensity lesions | Systemic manifestations, HLH, hypogammaglobinemia | Positive serology | CS, MTX, cyclosporin, etoposide, IVIG, HSCT |  |
| P34 | weeks et al., USA (2006) (31)  case report | 18 | 7 | 18 | Short-term memory disruption, episodic disorientation, and lethargy | Encephalitis, vasculitis, Burkitt lymphoma of terminal ileus, CNS lymphoma, recurrent URIs, herpes zoster infection, pneumonia | Elevated WBCs and protein | Extensive tumor necrosis, immune cell vascular infiltration, thrombosis | Multifocal vasculopathy with fusiform aneurysmal dilations | Systemic manifestations | Positive serology, EBV copies > 48000, positive tissue infection | IVIG, antimicrobials, CS, hydroxyurea, cyclosporin, RTX, mAb, HSCT | At 19 years old |
| P35 | Wu et al., China (2022) (32)  Case report | 5 | 4 yr 9 month | 4 yr 9 month | Septic pyemia | Fever, headache, lower extremities weakness, repeated hepatosplenomegaly | Elevated WBCs and protein |  | Subarachnoid and  cerebral hemorrhage, widespread inflammatory lesion | Systemic manifestations, HLH | Positive serology | Etoposide, busulfan, FLU, ATG, HSCT |  |
| P36 | Zhu et al., China (2013) (33)  Case report | 5 | 3 | 5 | Headache, vomiting, abnormal gait, obnubilation, generalized seizure | Vasculitis, Burkitt lymphoma | Elevated WBCs, protein, and low glucose |  | Cerebral edema, demyelination, multiple hypodense masses | Systemic manifestations | Negative serology | Mannitol, CS, antimicrobials |  |
| P37 | Ochiai, S., et al., Japan (2022) (34)  Case report | 12 | 12 | 12 | Seizures, behavioral changes, short term memory loss, non-convulsive status epilepticus | Encephalitis, interstitial pneumonia, limbic encephalitis |  |  | High signal intensities in white matter | Systemic manifestations, lymphoproliferation | Negative serology | CS, IVIG, PE, CYC, MMF, mAb, etoposide | At 12 years old |
| P38 | Kanegane, H. et al., Japan (35) (2005)  Case report | 22 | 3 | 22 | Memory disturbance | Vasculitis, acute EBV-induced minimal change disease, hypogammaglobinemia |  |  | Enhanced nodular lesions | Systemic manifestations, HLH, hypogammaglobinemia | Negative serology | CS |  |
| P39 | Dutz JP et al., Canada (2001) (36)  Case report | 12 | 13 months | 12 | Gradual blindness,  Left leg weakness,  mononeuritis | Vasculitis, chorioretinitis | Elevated RBCs | Vasculitis | Muscle atrophy and denervation | Systemic manifestations, HLH, hypogammaglobinemia | Negative serology, positive tissue infection |  | At 12 years old |
| P40 | Verhelst H et al (2007) (37)  Case report | 16 | 9 | 16 | decreased alertness, short-term memory disturbances,  generalized tonic clonic seizure,  limbic encephalitis | Vasculitis, B-cell non-Hodgkin lymphoma |  | Vasculitis | Bilaterally abnormal signals in the mesial temporal areas | Hypogammaglobinemia, lymphoproliferation |  | CS, anti-CD20*, MTX | At 17 years old |
| P41 | Parida, UK (2022) (38) case series | 4 | 2 | 2 | Irritability, aphasia, seizures,  developmental  delay | 0 | Elevated WBCs and protein | Perivasculitis | Bilateral white matter signal abnormalities,  severe  atrophy, gliosis, subdural effusion | Systemic manifestations, HLH |  | CS, IVIG, CYC, HSCT |  |
| P42 | This article,  Case report | 10 | 5 | 5 | Decreased consciousness, generalized tonic clonic seizures, truncal ataxia | Encephalitis, vasculitis, Burkitt lymphoma |  | Lymphoplasmacytic encephalitis | High-intensity cortical and basal ganglia lesions | Hypogammaglobinemia, lymphoproliferation | Negative serology, positive tissue infection | CS, RTX, HSCT | At 10 years old |

***** MRI = magnetic resonance imaging, EBV = Epstein–Barr virus, HSCT = hematopoietic stem cell transplantation, CS = corticosteroid, CYC = cyclophosphamide, HLH = Hemophagocytic lymphohistiocytosis, RTX = rituximab, WBC = white blood cell, MTX = methotrexate, rATG = rabbit anti-thymocyte globulin, MMF = mycophenolate mofetil, AZA = azathioprine, FLU = fludarabine, mAb = monoclonal antibody, IVIG = intravenous immunoglobulin, HCQ = hydroxychloroquine, IFN-γ = interferon gamma, PE = plasma exchange, URI = upper respiratory tract infection, MUD = matched unrelated donor, CD20 = cluster of differentiation 20

**References**

1. Blackburn PR, Lin WL, Miller DA, Lorenzo-Betancor O, Edwards ES, Zimmermann MT, et al. X-Linked Lymphoproliferative Syndrome Presenting as Adult-Onset Multi-Infarct Dementia. J Neuropathol Exp Neurol. 2019;78(5):460-6.

2. Bohne S, Kentouche K, Petersen I, Fritzenwanger M, Pletz MW, Lehmberg K, et al. Fulminant epstein-barr virus-associated hemophagocytic lymphohistiocytosis. The Laryngoscope. 2013;123(2):362-5.

3. Børresen ML, Lundstrøm KE, Ifversen M, Jennum PJ. Sodium oxybate (Xyrem) treatment in severely sleep-deprived child with Epstein-Barr virus encephalitis with lesion of sleep-wake regulation system: a case report. Sleep Med. 2019;62:29-31.

4. Chartier ME, Deheragoda M, Gattens M, Dhawan A, Heaton N, Booth C, Hadžić N. Successful Auxiliary Liver Transplant Followed by Hematopoietic Stem Cell Transplantation in X-Linked Lymphoproliferative Disease Type 1. Liver Transpl. 2021;27(3):450-5.

5. Escaron C, Ralph E, Bibi S, Visser J, Aricò M, Rao K, et al. Diagnosis of HLH: two siblings, two distinct genetic causes. Clinical and Experimental Immunology. 2022;207(2):205-7.

6. Ghosh T, Guerrero-Pena A, Kashyap M, Saad AG, Thorson CM, Pillai AB. Asymptomatic incidental neuroblastoma in a patient with SH2D1A deficiency. Pediatr Blood Cancer. 2022;69(1):e29314.

7. Goodyer M, Sargent J, Bond J, McMahon C, Dunne B, Smith O. Allogeneic stem cell transplantation as immunotherapy for X-linked lymphoproliferative disease-associated cerebral T-cell lymphoma. British Journal of Haematology. 2013;163(1):133-5.

8. Gray PE, O'Brien TA, Wagle M, Tangye SG, Palendira U, Roscioli T, et al. Cerebral Vasculitis in X-linked Lymphoproliferative Disease Cured by Matched Unrelated Cord Blood Transplant. J Clin Immunol. 2015;35(7):604-9.

9. Hervier B, Latour S, Loussouarn D, Rimbert M, De-Saint-Basile G, Picard C, Hamidou M. An atypical case of X-linked lymphoproliferative disease revealed as a late cerebral lymphoma. J Neuroimmunol. 2010;218(1-2):125-8.

10. Hügle B, Astigarraga I, Henter J-I, Porwit-MacDonald A, Meindl A, Schuster V. Simultaneous manifestation of fulminant infectious mononucleosis with haemophagocytic syndrome and B-cell lymphoma in X-linked lymphoproliferative disease. European Journal of Pediatrics. 2007;166(6):589-93.

11. Sheth J, Patel A, Shah R, Bhavsar R, Trivedi S, Sheth F. Rare cause of Hemophagocytic Lymphohistiocytosis due to mutation in PRF1 and SH2D1A genes in two children – a case report with a review. BMC Pediatrics. 2019;19(1):73.

12. Jiang Y, Firan M, Nandiwada SL, Reyes A, Marsh RA, Vogel TP, Hajjar J. The Natural History of X-Linked Lymphoproliferative Disease (XLP1): Lessons from a Long-Term Survivor. Case Reports Immunol. 2020;2020:8841571.

13. Karasawa T, Kudo K, Tanita K, Takahashi Y, Kanegane H, Terui K. Epstein-Barr Virus-Negative Granulomatous Disease Due to SAP Deficiency. J Clin Immunol. 2021;41(6):1372-5.

14. Korah-Sedgwick MM, Wall LA. EBV Infection in XLP1 Manifested Solely by Behavioral Aggression and Effective Treatment Using Rituximab. Case Reports in Immunology. 2018;2018(1):3705376.

15. Kusano N, Sakata N, Sugimoto K, Miyazawa T, Ueda S, Okano M, et al. An 18-Year-Old Male With X-linked Lymphoproliferative Syndrome Type 1 Who Developed Primary Central Nervous System Lymphoma 6 Months After Primary Epstein-Barr Virus Infection. J Pediatr Hematol Oncol. 2019;41(8):e538-e41.

16. Kwon WK, Kim JA, Park J-H, Kim DR, Park SE, Kim YJ, et al. Case Report: Novel Splicing Variant in SH2D1A in a Patient With X-Linked Lymphoproliferative Syndrome Type 1. Frontiers in Pediatrics. 2022;Volume 10 - 2022.

17. Li B, Chen W, Cai X, Hai Y, Pang Q, Xiang W, Zhang Z. Case report: Non-EBV associated cerebral vasculitis and cerebral hemorrhage in X-linked lymphoproliferative disease. Front Immunol. 2024;15:1381472.

18. Liu J, Tian W, Wang F, Teng W, Zhang Y, Tong C, et al. Maternal onset de novo SH2D1A mutation and lymphocytic choriomeningitis virus infection in a patient with X‑linked lymphoproliferative disease type 1: A case report. Mol Med Rep. 2015;11(5):3291-4.

19. Mejstríková E, Janda A, Hrusák O, Bucková H, Vlcková M, Hancárová M, et al. Skin lesions in a boy with X-linked lymphoproliferative disorder: comparison of 5 SH2D1A deletion cases. Pediatrics. 2012;129(2):e523-8.

20. Mukai T, Waki K. X-linked lymphoproliferative syndrome associated with Epstein–Barr virus encephalitis and lymphoproliferative disorder. Clinical Case Reports. 2023;11(9):e7949.

21. Nademi Z, Radwan N, Rao K, Gilmour K, Worth A, Booth C. Different Phenotypic Presentations of X-Linked Lymphoproliferative Disease in Siblings with Identical Mutations. J Clin Immunol. 2019;39(5):523-6.

22. Nallasamy S, Eagle RC, Jr., Rorke-Adams L, Nichols KE, Brucker AJ. Eye findings in X-linked lymphoproliferative disorder. Retina. 2011;31(4):790-7.

23. Neves JF, Raga LT, Chiang SCC, Tesi B, Vieira JP, Cordeiro AI, et al. Fatal Central Nervous System Lymphocytic Vasculitis after Treatment for Burkitt Lymphoma in a Patient with a SH2D1A Mutation. Pediatr Infect Dis J. 2019;38(2):e29-e31.

24. Ortega C, Estévez Orlando A, Fernández S, Aguado R, Rumbao José M, Gonzalez T, et al. Interleukin-21 Overexpression Dominates T Cell Response to Epstein-Barr Virus in a Fatal Case of X-Linked Lymphoproliferative Syndrome Type 1. Clinical and Vaccine Immunology. 2013;20(5):765-71.

25. Sankararaman S, Riel-Romero RM, Jeroudi M, Gonzalez-Toledo E. Epstein-Barr virus induced hemophagocytic lymphohistiocytosis in X-linked lymphoproliferative disease. J Neurosci Rural Pract. 2014;5(2):171-4.

26. Sperl D, Benesch M, Urban C, Lackner H, Sovinz P, Speicher MR, et al. Fatal EBV infection and variable clinical manifestations in an XLP-1 pedigree - rapid diagnosis of primary immunodeficiencies may save lives. Klin Padiatr. 2012;224(6):386-9.

27. Steininger J, Rossmanith R, Geier CB, Leiss-Piller A, Thonhauser L, Weiss S, et al. Case Report: Meningoencephalitis With Thrombotic Occlusive Vasculopathy in a Young EBV-Naïve Boy Is Associated With a Novel SH2D1A Mutation. Frontiers in Immunology. 2021;12.

28. Talaat KR, Rothman JA, Cohen JI, Santi M, Choi JK, Guzman M, et al. Lymphocytic vasculitis involving the central nervous system occurs in patients with X-linked lymphoproliferative disease in the absence of Epstein-Barr virus infection. Pediatr Blood Cancer. 2009;53(6):1120-3.

29. Trottestam H, Beutel K, Meeths M, Carlsen N, Heilmann C, Pašić S, et al. Treatment of the X-linked lymphoproliferative, Griscelli and Chédiak–Higashi syndromes by HLH directed therapy. Pediatric Blood & Cancer. 2009;52(2):268-72.

30. Voeten M, Maes P, Wojciechowski M, Vandenbossche L, Meyts I, Ceulemans B. Extremely elevated cerebrospinal fluid protein levels in a child with neurologic symptoms: beware of haemophagocytic lymphohistiocytosis. Eur J Paediatr Neurol. 2014;18(3):427-9.

31. Weeks JK, Helton KJ, Conley ME, Onciu M, Khan RB. Diffuse CNS vasculopathy with chronic Epstein-Barr virus infection in X-linked lymphoproliferative disease. AJNR Am J Neuroradiol. 2006;27(4):884-6.

32. Wu L, Yang F, Wang J, Yang F, Liang M, Yang H. Exon skipping caused by a complex structural variation in SH2D1A resulted in X-linked lymphoproliferative syndrome type 1. Mol Genet Genomic Med. 2022;10(3):e1873.

33. Zhu J, Zhang Y, Zhen ZJ, Chen Y, Wang J, Cai RQ, Sun XF. Lymphoma and cerebral vasculitis in association with X-linked lymphoproliferative disease. Chin J Cancer. 2013;32(12):673-7.

34. Ochiai S, Hayakawa I, Ohashi E, Hamano S, Miyata Y, Sakuma H, et al. Fatal X-linked lymphoproliferative disease type 1-associated limbic encephalitis with positive anti-alpha-amino-3-hydroxy-5-methyl-4-isoxazolepropionic acid receptor antibody. Brain and Development. 2022;44(9):630-4.

35. Kanegane H, Ito Y, Ohshima K, Shichijo T, Tomimasu K, Nomura K, et al. X-linked lymphoproliferative syndrome presenting with systemic lymphocytic vasculitis. American Journal of Hematology. 2005;78(2):130-3.

36. Dutz JP, Benoit L, Wang X, Demetrick DJ, Junker A, de Sa D, Tan R. Lymphocytic vasculitis in X-linked lymphoproliferative disease. Blood. 2001;97(1):95-100.

37. Verhelst H, Van Coster R, Bockaert N, Laureys G, Latour S, Fischer A, Haerynck F. LIMBIC ENCEPHALITIS AS PRESENTATION OF A SAP DEFICIENCY. Neurology. 2007;69(2):218-9.

38. Parida A, Abdel-Mannan O, Mankad K, Foster K, Ramdas S, Ram D, et al. Isolated central nervous system familial hemophagocytic lymphohistiocytosis (fHLH) presenting as a mimic of demyelination in children. Multiple Sclerosis Journal. 2021;28(4):669-75.
