# Supplementary material for: The neurologic face of X-linked lymphoproliferative syndrome type 1: a systematic review
Source: Orphanet J Rare Dis. 2025 Oct 21;20:528. doi: 10.1186/s13023-025-04057-9 (PMC12541938; doi:10.1186/s13023-025-04057-9)
Supplement: Supplementary file 5 — Supplementary Material 5 [file 13023_2025_4057_MOESM5_ESM.docx]

| **Patient** | **Mutation** | **Protein Change** | **Ref** |
| --- | --- | --- | --- |
| P1 | c.35G>T | p.Ser12Ile | Blackburn, P. R., et al. 2019 |
| P2 | Non-specified mutation |  | Bohne, S., et al. 2013 |
| P3 | whole gene deletion on chromosome X |  | Børresen, M. L., et al. 2019 |
| P4 | c.245dupT | p.Asn82Lysfs*22 | Chartier, M.-E., et al. 2021 |
| P5 | c.301C>A | p.Pro101Thr | Escaron, C., et al. 2022 |
| P6 | c.385T>G | p.Tyr129Asp | Ghosh, T., et al. 2022 |
| P7 | deletion of the entire *SH2D1A* gene with absent SAP expression |  | Goodyer, M., et al. 2013 |
| P8 | c.96G>C | p.Trp32Cys | Gray, P. E., et al. 2015 |
| P9 | c.163C>T | p.Arg55* | Hervier, B., et al. 2010 |
| P10 | c.146insG | p.Gly49fs*18 | Hügle, B., et al. 2007 |
| P11 | c.138-3C>G | p.Arg47Glyfs*34 | J, S., et al. 2019 |
| P12 | Full deletion of SH2D1A |  | Jiang, Y., et al. 2020 |
| P13 | deletion of exon 2 of the *SH2D1A* |  | Karasawa, T., et al. 2021 |
| P14 | c.191G>A | p.Trp64* | Korah-Sedgwick, M., et al. 2018 |
| P15 | c.208_209insC | p.Thr70Hisfs* | Kusano, N., et al. 2019 |
| P16 | c.162_201+31delinsTACAAGGACATATACA, exon 2 deletion |  | Kwon, W. K., et al. 2022 |
| P17 | c.192G>A | p.Trp64* | Li, B., et al. 2024 |
| P18 | c.7G>T  c.228T>A | p.Ala3Ser  p.Tyr76* | Liu et al., ,2015 |
| P19 | SH2D1A deletion |  | Mejstríková et al. 2012 |
| P20 | c.239_240insA | p.Lys80fs*22 | Mukai et al., 2023 |
| P21 | deletions of exon 2 of the *SH2D1A* |  | Nademi et al. , 2019 |
| P22 | c.163C>T | p.Arg55* | Nallasamy et al, 2011 |
| P23 | c.201G>A | p.Glu67Glu | Neves et al. , 2019 |
| P24 | exon 2 of the SH2D1A gene, the nonsense mutation | p.Arg55* | Ortega et al., 2013 |
| P25 | reduced expression of SLAM and confirmed genetic mutation in the *SH2D1A* |  | Sankararaman et al., 2014 |
| P26 | c.20A>C | p.Tyr7Cys | Sperl et al., 2012 |
| P27 | c.20A>C | p.Tyr7Cys | Sperl et al., 2012 |
| P28 | c.2T>G | p.M1Thr | Steininger et al., 2021 |
| P29 | c.163C>T | p.Arg55* | Talaat et al., 2009 |
| P30 | c.163C>T | p.Arg55* | Talaat et al., 2009 |
| P31 | sap mutation |  | Trottestam et al., 2009 |
| P32 | sap mutation |  | Trottestam et al., 2009 |
| P33 | c.164G>A | p. Arg55Gln | Voeten et al., 2014 |
| P34 | deletion of exon 1 of *SH2D1A* |  | weeks et al., 2006 |
| P35 | Breakpoints in exon 2 of *SH2D1A* and intron 2 |  | Wu et al., 2022 |
| P36 | a C＞T nonsense substitution mutation |  | Zhu et al., 2013 |
| P37 | c.192G>A | p.Trp64* | Ochiai, S., et al. 2022 |
| P38 | c.321C>G | p.His8Asp | Hoshino, T. et al 2005 |
| P39 | G to T exon 2 | p.Arg55Leu | Dutz JP et al 2001 |
| P40 | No mutation found. However, deficiency in SAP protein expression was confirmed by immunoblotting |  | Verhelst H et al 2007 |
| P41 | Non-specified mutation |  | Parida, et al., 2022 |
| P42 | c.192 G>A | p.Trp64* | This article |

Supplementary Table 5: Genetic findings of the patients (*SH2D1A* Ref number: ENST00000371139.9)
